# Bioinformatics insights into acute lung injury/acute respiratory distress syndrome

**DOI:** 10.1186/2001-1326-1-9

**Published:** 2012-06-09

**Authors:** Xiaocong Fang, Chunxue Bai, Xiangdong Wang

**Affiliations:** 1 Department of Pulmonary MedicineZhongshan Hospital, Fudan University, Shanghai, China; 2 Biomedical Research Center, Zhongshan Hospital, Fudan University, Shanghai, China; 3 Institute of Clinical Science, Lund University, Lund, Sweden

**Keywords:** Acute lung injury, Acute respiratory distress syndrome, Genomics, Proteomics, Metabolomics, Bioinformatics

## Abstract

Bioinformatics is the application of omics science, information technology, mathematics and statistics in the field of biomarker detection. Clinical bioinformatics can be applied for identification and validation of new biomarkers to improve current methods of monitoring disease activity and identify new therapeutic targets. Acute lung injurt (ALI)/Acute respiratory distress syndrome (ARDS) affects a large number of patients with a poor prognosis. The present review mainly focused on the progress in understanding disease heterogeneity through the use of evolving biological, genomic, and genetic approaches and the role of clinical bioinformatics in the pathogenesis and treatment of ALI/ARDS. The remarkable advances in clinical bioinformatics can be a new way for understanding disease pathogenesis, diagnosis and treatment.

## Review

### Introduction

In recent years, there is a great increase in genomics and other molecular research which produced a tremendous amount of information related to molecular biology [1-3]. Meantime, various powerful data mining and statistical bioinformatics methods have been propagated for identifying, prioritizing and classifying robust and generalizable biomarkers with high discriminatory ability [4], and some online bioinformatics data libraries, such as Enzyme, KEGG, Gene Ontology, NCBI Taxonomy, SwissProt and TrEMBL were generated for the store and management of data. Bioinformatics now entails the creation and advancement of databases, algorithms, computational and statistical techniques and theory to solve formal and practical problems arising from the management and analysis of biological data.

**Figure 1:**
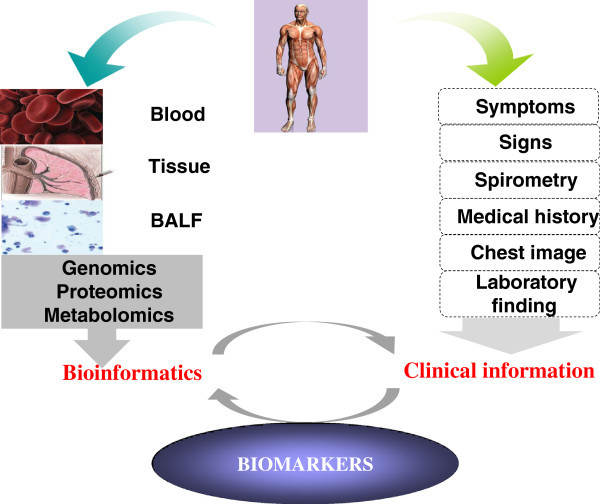
**The optimum pattern of clinical bioinformatics in ALI/ARDS.** We get clinical information including symptoms, physical signs, medical history, lung function or images from the patients. On the other hand, we draw bioinformation from their blood, BALF or tissue. Then, the clinical information and bioinformation should be validated and complemented with each other in order to detect effective and powerful biomarkers. Only in this way, the bioinformatics technologies can be helpful.

Clinical bioinformatics is a new emerging science combining clinical information, (including patients’ complaints, history, clinical symptoms and signs, physicians’ examinations, biochemical analysis, imaging profiles, pathologies), omics science, mathematics, information technology and library data together. It may play a potentially important role in the discovery of biomarkers, which will facilitate the diagnosis of disease, as well as treatment. A commonly used definition of a biomarker is “a characteristic that is objectively measured and evaluated as an indicator of normal biological process, pathogenic processes, or pharmacologic responses to a therapeutic intervention” [5]. Clinical bioinformatics is also a new way to focus on the combination of clinical symptoms and signs with human tissue-generated bioinformatics to get deep and full understand of the risk factors, pathogenesis and progress of human disease. Recent technological developments in genomics, proteomics, metabolomics, are allowing researchers to take an unbiased ‘big-picture’ approach to further elucidate the molecular pathogenesis of lung injury [6] (Figure 1). Recent years, clinical bioinformatics have been widely applied in the investigation of various kinds of diseases including acute lung injury.

Acute lung injury (ALI)/acute respiratory distress syndrome (ARDS) remains a major cause of high morbidity, mortality, and health care costs in critically ill patients [7]. ALI/ARDS is an acute life-threatening form of hypoxemic respiratory failure, characterized by lung tissue edema and injury, inflammatory responses, and compromised gas exchange following macrophage activation, surfactant dysfunction, and epithelial destruction. Inflammatory cells could be activated to produce a large number of mediators in the early stage of the disease, resulting in injury to both the vascular endothelium and the alveolar epithelium [8,9]. Injury to the alveolar-capillary barrier increases permeability of the alveolar wall, increases accumulation of inflammatory cells and cytokines, and impairs alveolar fluid clearance, resulting in accumulation of protein-rich edema in the alveolar space [9].

The incidence of ALI/ARDS was reported to be 80 per 100,000 person-years with a mortality rate of 40% in United States [10]. A prospective study conducted in Chinese pediatric intensive care units indicated that the incidence of ARDS in critically ill patients was 2.7% with a mortality rate of 44.8% [11]. The reported incidence of pediatric ARDS in US was about 9.5% [12], with the overall mortality rate of 22% [13]. Over the past two decades, a variety of interventions and intensive care strategies have been used in treating patients with ALI/ARDS. However, only low tidal volume ventilation accompanied with fluid management has been shown to be beneficial in improving morbidity and mortality [14]. There is an urgent need to identify and validate disease-specific biomarkers and novel therapeutic strategies for ALI/ARDS.

### Application of bioinformatics in ALI/ARDS

Genomics study in ALI is an approach to detect associated genetic variations, or gene polymorphisms, which may contribute to ALI susceptibility, severity or prognosis in a racial- and ethnic-specific manner. Examples of this approach include microarray based genome-wide expression analyses [15] and genotype detection for specific gene [16]. Although genome-wide expression profiling is an excellent method for generating long lists of candidates, it is proving to be more difficult to develop follow-on approaches to select critical genes. Then, if specific gene is supposed to be involved in the pathogenesis of ALI/ARDS, genotyping by PCR or sequencing can be applied to validate the significance of those suspected genes. Proteomics captures a comprehensive set of expressed proteins in a cell or organism to understand biological function of cells or organs [17-19]. Commonly used methods for current proteomics studies include two-dimensional gel electrophoresis (2-DE) followed by matrix-assisted laser desorption/ionization time-of-flight mass spectrometry (MALDI-TOF MS), liquid chromatography coupled to electrospray ionization tandem MS (LC-ESI MS/MS), surface-enhanced laser desorption/ionization coupled to TOF MS (SELDI-TOF MS), capillary electrophoresis coupled to MS, and microarrays [19]. In the pulmonary proteomic studies, bronchoalveolar lavage fluid (BALF), lung tissue, blood and alveolar cells have been used extensively.

The collection of global metabolic data and their interpretation (both spectral and biochemical) with modern spectroscopic techniques and appropriate statistical approaches is called “metabolic profiling” or “metabolomics” [20-22]. It is an emerging component of the systems biology approach for the discovery of clinically relevant biomarkers and potential therapeutic targets [23]. The most often applied technology is nuclear magnetic resonance (NMR) and mass spectrometry [24-26]. Since NMR spectrometry can identify and quantify multiple metabolites from a single liquid or solid sample and it’s cost effective and robust, it proved to be very useful in metabolomics studies.

### Biomarker development in ALI/ARDS

#### Genomics

There are several publications reviewed the application of genomics in the study of ALI. In 2002, Marshall and his colleagues provide the first evidence of a genetic influence in ARDS, suggesting an important role for angiotensin converting enzyme (ACE) [16]. In this study, a total of 358 patients were enrolled including 96 patients fulfilling American/European Consensus Committee criteria for ARDS, 174 coronary artery bypass graft (CABG) controls and 88 non-ARDS but require mechanical ventilation patients in the intensive care unit (ICU). DNA was extracted from whole blood samples and ACE genotype was determined by three-primer polymerase chain reaction amplification. This study detected that the frequency of the DD genotype and the D allele was markedly increased in patients with ARDS compared with both the CABG and ICU comparison groups and the healthy population sample [16].

Similarly, Gong, et al demonstrated that -308GA promoter polymorphism in the TNF-a gene was associated with the mortality of ARDS [27]. SNPs of 43 Toll like receptor-related genes were identified and one of these genes was demonstrated to be associated with susceptibility to organ dysfunction and death in patients with sepsis [28]. Later, pre–B-cell colony-enhancing factor (PBEF) was found significantly increased in the expression profiling in animal models of ALI and in human ALI. In this study, multiple technologies including PCR, microarray analysis and some proteomic measurement methods were used. This combination of various technologies in bioinformatics study is important and helpful [29].

However, different from these traditional epidemiological studies in at risk populations, Marzec.et al first applied positional cloning and gene sequencing to identify candidate susceptibility genes and SNP polymorphism. They demonstrated that the -617 A SNP of Nrf2 (NF-E2 related factor 2) had a significantly higher risk for developing ALI after major trauma [30]. Then a large-scale candidate gene genotypings were carried out in a cohort of critically ill subjects with trauma to identify ALI risk variants after major trauma. Five SNPs were found to have a significant association with ALI, of which two SNPs in ANGPT2 (rs1868554 and rs2442598) replicated the significant association with ALI. And furthermore, the top associated SNPs were tested in a multicenter case-control population [31]. In a two-stage discovery/validation study, the commonly occurring FAS haplotype including FAS21541T and FAS9325A was significantly associated with ALI susceptibility and increased FAS mRNA expression in peripheral leukocytes in response to innate immune stimulation [32]. A complete summary of all published applications of genetics in the study of ALI/ARDS is far beyond the scope of this review. Table 1 provides a summary, with references, of some of the most recently or interesting applications of genetics in the diagnosis, susceptibility or prognosis of ALI/ARDS [31-37].

**Table 1:** Examples of application for genetics in the pathogenisis of ALI in 2011

**Genes**	**Technologies**	**Relationship with ALI**	**Ref.**
NADPH oxidase	NOX2 subunit knockout mice	NOX-2(-/-) mice exhibited diminished TNF alpha-induced acute inflammatory responses in the lungs but not other tissues, as evidenced by decreased activation of the redox-sensitive transcription factor NF-kappa B, and decreased gene expression of IL-1 beta, IL-6, TNF alpha, E-selectin, and other cellular adhesion molecules.	[33]
ANGPT	SNP array performed in ALI and non-ALI patients	An ANGPT2 region is associated with both ALI and variation in plasma angiopoietin-2 isoforms.	[31]
Fas (CD95)	Genptyped 14 SNPs in FAS in healthy white volunteers and patients with ALI	Common genetic variants in FAS are associated with ALI susceptibility	[32]
Surfactant protein B (SPB)	Genotyping was performed on seven linkage isequilibrium-tag SNP in the surfactant protein B gene	SPB are associated with more severe lung injury as indicated by the association of specific SNP genotypes and haplotypes with the need for mechanical ventilation in African American children with community-acquired pneumonia.	[34]
Acvr1, Arhgap15, Cacnb4, Cacybp, Ccdc148,Fancl, Mycn, Mgat4a, Rfwd2, Tgfbr3, and Tnn	haplotype association mapping, microarray/qRT-PCR analyses, in silico SNP	11 candidate genes are associated with acrolein-induced acute lung injury in 40 different inbred strains of mice	[35]
IRAKs	tagging SNPs array	common SNPs in IRAK3 gene might be determinants for sepsis-induced ALI. association with ALI development among septic patients	[36]
nmMLCK	nmMLCK knockout mice, nmMLCK silencing RNA	nmMLCK knockout mice were significantly protected from VILI,	[37]

Abbreviations: *Ref*:Reference; *ANGPT*:angiopoietin; *NADPH*:glyceraldehyde-3-phosphate dehydrogenase; *NOX:NADPH* oxidases; *TNF*-alpha:tumor necrosis factor alpha; *IL-1*:interleukin-1; *IL-6*:interleukin-6; *SPB*:Surfactant protein B; *Acvr1*: activin A receptor, type 1; *ARHGAP15*:Rho GTPase activating protein 15; *CACNB4*:calcium channel, voltage-dependent, β 4 subunit; *CACYBP*:calcyclin binding protein; *CCDC148*:coiled-coil domain containing 148; *FANCL*: Fanconi anemia, complementation group L; *MYCN*:v-myc myelocytomatosis viral related oncogene, neuroblastoma derived (avian); *MGAT4A*: mannoside acetylglucosaminyltransferase 4, isoenzyme A; *RBMSl*:RNA binding motif, single stranded interacting protein 1; *RFWD2*:Ring finger and WD repeat domain 2; *TGFBR3*:transforming growth factor-β receptor III; *TNN*: tenascin N; *IRAKs*: interleukin-1 receptor-associated kinase genes; *nmMLCK*: non-muscle myosin light chain kinase isoform; *VILI*:ventilator induced lung injury.

Among these identified genes, some of them were well demonstrated to play critical roles in the development of ALI/ARDS. NADPH oxidase contributed to the production of reactive oxygen species, which is able to induce a cell death response in oxidative stress. NADPH oxidase 1 was reported to act as an important mediator of acute lung injury mediated by oxidative stress, particularly in alveolar cells. NOX1-generated ROS are largely responsible for alveolar cell death and subsequent lung injury through JNK and ERK pathways [38]. Angiopoietin-2 (ANGPT2) has been implicated in pulmonary vascular leak syndromes including ALI and sepsis in both animal and human studies [39,40], while recently, two *ANGPT2* SNPs (rs2442598 and rs1868554) were found to be strongly associated with the development of ALI in patients with major trauma [31]. However, studies are warranted to further elucidate the characterization of *ANGPT2* genetic variation and expression. Fas pathway has been investigated as a potential contributor to the inflammation and alveolar epithelial cell apoptosis observed in the lungs of patients with ALI [41,42]. SP-B enhances the rate of phospholipid absorption to the alveolar air/water interface [43], decreases surface tension by interfering with the attractive forces acting between water molecules [44] and has anti-inflammatory properties [45]. However, the others were seldom reported and futher studies are needed to validate their functions in ALI/ARDS.

Gene expression profiles represent only a small fraction of the complexity in an organism. On the other hand, gene expression data obtained by the microarray analysis are usually moderately correlated with the expression levels of corresponding protein products due to the post-transcriptional regulation. Thus, extrapolation of protein expression on the basis of microarray-based gene expression measurements must be validated by direct measurement of the protein [15]. Then proteomics comes.

#### Proteomics

Proteomics captures a nearly comprehensive set of expressed proteins in a cell or organism, which determine how a cell or organism functions. Proteomics become more practical and feasible for researchers in the field of pulmonary medicine using BALF, lung tissue, and exhaled breath condensates as the source of protein analysis [46]. In 2003, Bowler et al first applied the proteomic approach in the BALF, plasma and lung edema fluid in ALI/ARDS. In this paper, the author reported that although many of the proteins were demonstrated to have increased or decreased relative intensity in the plasma and EF of ALI patients compared with normal subjects, a limitation to the 2-DE proteomics approach is that it is not easy to quantify all changes in relative protein expression among a large number of samples. The author mentioned that the proteomics approach may be complementary to microarray studies [47].

Shotgun proteomics in BALF was analyzed and 870 different proteins were identified, including surfactant, proteases, and serum proteins. However, because of the several limitations of the 2DE method to detect particular classes of proteins [48], it can only be treated as an excellent screening tool to initially characterize a sample of mostly unknown protein composition, and additional methods, including isolation of subpopulations of proteins and further refinement of the methodologies must be cooperated to identify specific proteins [49]. Then, through the combined application of SELDI-TOF, 2-DE and western blot analysis, apolipoprotein A1, and S100 calcium-binding proteins A8 and A9 were detected to increase in the inflammatory BALF [50].

Following these studies, quantitative proteomic analysis was then used to profile the changes in protein expression in the lungs at the onset and during the course of acute lung injury. Computational analysis as used to map complex protein interactions in the lung fluids and study how these interactions changed during the course of ARDS. This approach to protein network analysis identified novel mediators of acute lung injury and protein pathways were redundant and involved in multiple biological processes [51].

**Figure 2:**
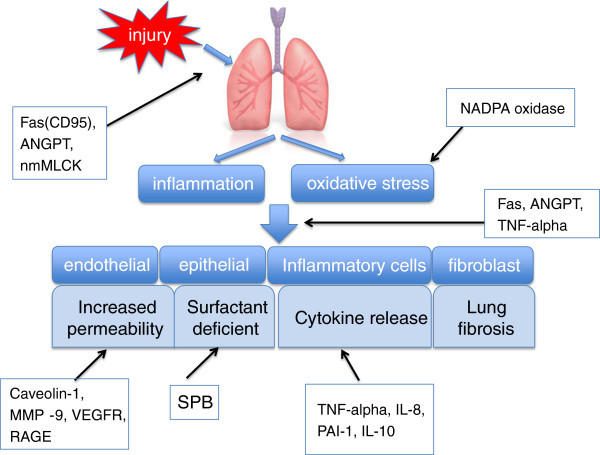
The potential mechanisms of these identified genes or proteins in the process of ALI/ARDS.

Table 2 provides a summary of the proteomic study in ALI/ARDS from 2009-2011 [52-58]. Caveolin-1 is involved in PMN adhesion, chemotaxis, and epithelial and endothelial cell apoptosis/senescence which accelerated lung injury in the early stage. However, cav-1 may be beneficial in the late stage through anti-fibrosis [59]. MMPs are a family of zinc-dependent proteinases which expressed by all cell types relevant to ARDS pathogenesis including alveolar epithelial cells, PMNs, macrophages, and fibroblasts. Many studies implicate MMPs in the injurious process as well ALI/ARDS, which may mainly contribute to its capability of degrading all components of the extracellular matrix [60,61]. Vascular endothelial growth factor (VEGF) and its receptors are critical in the regulation of both vascular permeability and endothelial cell survival. However, the role of VEGF and related molecules in ALI/ARDS is controversial [62]. PAI-1 is an endogenous inhibitor of the plasminogen activators. Studies reported that elevated level of PAI-1 is an independent risk factor for mortality and adverse clinical outcomes for ALI patient [63]. RAGE is a multi-ligand-binding receptor that can bind advanced glycation end products, amyloid β-peptide, S100 proteins, and high-mobility group box-1. RAGE-ligand interaction results in intracellular signaling, which leads to activation of the proinflammatory transcription factor nuclear factor-κB (NF-κB) [64,65]. Study indicated that RAGE may be useful as a biological marker of alveolar type I cell injury [66]. TNF-alpha and IL-8 are also well-validated inflammatory cytokines in ALI/ARDS [57]. Figure 2 integrated these factors together to show their potential roles in the pathogenesis of ALI/ARDS.

**Table 2:** Examples of application for proteomics in the pathogenisis of ALI in 2009-2010

**Protein**	**technologies**		**Ref.**
caveolin-1, CD44, annexin A2, protein S100-A10, or filamin A/B	Quantitative proteomic analysis	HMW-HA, via CD44-mediated CEM signaling events, represents a potentially useful therapeutic agent for LPS induced increasing of lung vascular permeability	[52]
MMP-9		Administration of doxycycline might be a significant supportive therapeutic strategy in prevention of VILI.	[53]
MMP-3, TIMP-1	liquid chromatography- electrospray ionization mass spectrometry	MMPs in the BALF of premature neonates maybe related to BPD development	[54]
VEGFR1, VEGFR2, NRP-1	Immunohistochemistry	VEGFR I and VEGFR2 were significant up-regulated in early ARDS while Neuropilin-1 was down-regulated.	[55]
PAI-1	ELISA	Higher PAI-1 levels are associated with increased mortality and fewer ventilator-free days among pediatric patients with ALI.	[56]
RAGE, PCPIII, BNP, ANG2, IL10, TNF-[alpha], IL8	bead-based cytokine array, singleplex enzyme-linked immunosorbent assay	seven plasma biomarkers had a high diagnostic accuracy in differentiating patients with trauma-induced ALI	[57]
AFT3	Microarray analysis, ELISA	ATF3 may act to counterbalance CS and high volume induced inflammation in Ventilator-induced Lung Injury	[58]

*HMW-HA*:high-molecular-weight hyaluronan; *CEM*: caveolin-enriched microdomains; *MMP*:matrix metalloproteinase; *TIMP*:tissue inhibitors of metalloproteinases; *VILI*:ventilation-induced lung injury; *BPD*:bronchopulmonary dysplasia; *GAPDH*:glyceraldehyde-3-phosphate dehydrogenase; *VEGF*:vascular endothelial growth factor; *NRP-1*:neuropilin-1; *RAGE*:receptor for advanced glycation end products; *PCPIII*:procollagen peptide III; *BNP*:brain natriuretic peptide; *ANG2*:angiopoietin-2; *IL-10*:interleukin-10, *TNF*-alpha:tumor necrosis factor alpha; *IL-8*:interleukin-8.

#### Metabolomics

Although the amplification of quantitative metabolomics in clinic bioinformaticsis in its infancy, it has been increasingly used recently. It is a unique tool of discovery because it identifies multiple endogenous markers, and it changes over time which can be predictive of clinical outcome and therapeutic response [67,68]. The ability of magnetic resonance imaging (MRI) and metabolic NMR were investigated to detect and quantify inflammation-mediated lung injury [69]. Recently, proton nuclear magnetic resonance ^(1^ H-NMR) based quantitative metabolomics with subsequent computational analysis was applied to investigate the plasma metabolic changes associated with sepsis-induced ALI. Several metabolites including glutathione, adenosine, phosphatidylserine, and sphingomyelin were found different between patients with ALI and healthy subjects. Additionally, computational network analysis was carried out to investigate a distinct metabolic pathway for each metabolite [24]. Moreover, there is another case-control study conducted on plasma samples from six albumin-treated and six saline-treated patients to measure the metabolic changes after albumin treatment. This study showed that blood albumin levels normalized and oxygenation improved with albumin treatment compared to saline treatment. In this study, 1 H-NMR spectroscopy was introduced to characterize systemic metabolic responses and identify patients’ unique response to pharmacologic therapy and appealed to play a potential role in the guiding treatment and reducing healthcare cost [68].

In summary, ALI/ARDS is a complex disease which can be initiated by a diverse array of precipitating factors and involving the interplay of both the environment and genetic factors. However, numerous environmental agents can induce common pathological changes, including damage to endothelial and epithelial cells, infiltration of inflammatory cells, cytokine release and so on. Those common biological pathways may be controlling generalized responses to injury and repair which cannot distinguish the type of lung injury across different models. On the other hand, susceptibility to ALI/ARDS is also different among patients with same conditions. Clinical models have identified risk factors and laboratory testing to evaluate severity, guide clinical therapy and tell prognosis. However, these strategies remain imprecise. The application of bioinformatics enables us to identify the individual expression patterns as well as the injury-specific pattern, thus may facilitate therapy and predict prognosis. Besides, in the absence of significant insights into disease pathogenesis, comprehensive bioinformatics technologies may help us to get deep understanding of such complex and life-threatening illness thus may provide novel therapeutic targets. Finally, there are several available samples that can be used in the bioinformatics study of the lung, such as exhaled breath gas, blood, lung tissue, pleural effusion and BALF, which may largely facilitate the biomarker research.

## Conclusion

The genetic and proteomic approach to study ALI/ARDS in clinical setting is still in an early stage, while some important data have been generated. There is a great need of further exploratory studies to better understand the molecular mechanisms and pathophysiology of ALI/ARDS. Much effort is also needed to validate whether these biomarkers and drug targets are really useful in early detection, prediction, and so are prevention and treatment of lung diseases. The most important issue to apply clinical bioinformation for target and biomarker identification and validation should to integrate bioinformatics from genomics, proteomics and metabolomics with clinical informatics and findings. We should also think the value and role of clinical bioinformatics in clinical research and study on ALI/ARDS to benefit personalized medicine and patient prognosis.

Different from other informatics, clinical bioinformatics should focus more on clinical informatics, including patient complaints, history, therapies, clinical symptoms and signs, physician's examinations, biochemical analyses, imaging profiles, pathologies and other measurements [70]. For example, a recent study developed a digital evaluation scoring system to translate disease complexity of patient information, clinical data, standard laboratory evaluations, imaging data and symptoms into the digital [71]. This particular study tried to combine clinical informatics with protein profiles generated from antibody arrays to evaluate the disease-specific, severity-associated, duration-related biomarkers. Although the understanding of bioinformatics, clinical bioinformatics, and clinical informatics remains limited among clinicians, we believe clinical bioinformatics will have a significant impact on critical care medicine and public health. Since clinical bioinformatics is a multidisciplinary field, which combined clinical informatics, omics science, information technology, mathematics and statistics together, we suggest the cooperation of clinicians and technologists in this field. Progress in understanding of disease heterogeneity through the use of evolving biological, genomic, and genetic approaches should provide major new insights into the pathogenesis and treatment of ALI. Any effective and novel intervention generated from clinical bioinformatics may prevent or improve the high mortality and morbidity of ALI/ARDS.

## Competing interests

The authors declare that they have no competing interests.

## Authors’ contributions

XF contribute to collection of information, analysis and interpretation of data and writing of the manuscript. CB: contribute to revision of the manuscript. XW: contribute to the critical revision of the manuscript. All authors read and approved the final manuscript.
